# Supplementary material for: Protein Intake, Metabolic Status and the Gut Microbiota in Different Ethnicities: Results from Two Independent Cohorts
Source: Nutrients. 2021 Sep 10;13(9):3159. doi: 10.3390/nu13093159 (PMC8465773; doi:10.3390/nu13093159)
Supplement: Supplementary file 1 [file nutrients-13-03159-s001.zip › supplementary/Supplementary Figure S2. Forrest plots-BMI representing protein intake in the A) MetaCardis and B )HELIUS cohort.pdf]

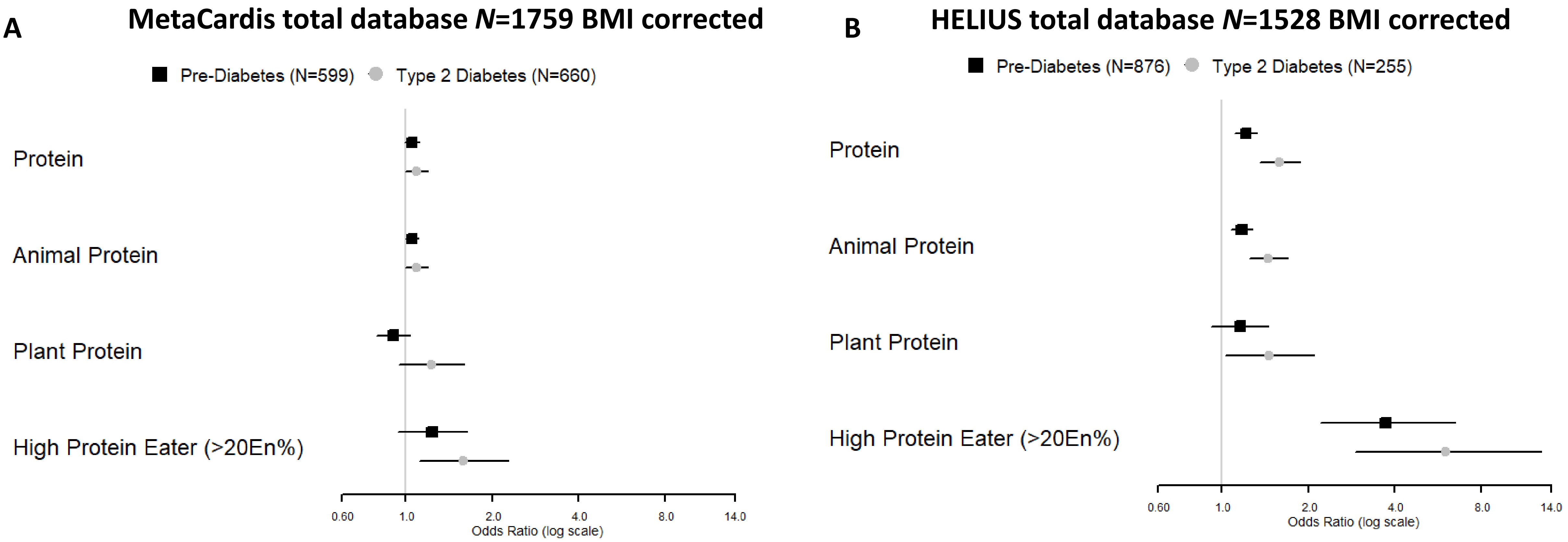

Supplementary Figure S2: Forrest plots representing protein intake in the A) MetaCardis and B) HELIUS cohort. Data is shown per 10gr of protein intake and the model was adjusted for age, gender, physical activity, BMI, other macronutrients and total energy intake according to the energy residual model.

Model used:

Metabolic status=protein_residual+carbohydrate_residual+fat_residual+fibre_intake+total_kcal_intake+ age+gender+physical_activity+BMI
