# Supplementary material for: Protein Intake, Metabolic Status and the Gut Microbiota in Different Ethnicities: Results from Two Independent Cohorts
Source: Nutrients. 2021 Sep 10;13(9):3159. doi: 10.3390/nu13093159 (PMC8465773; doi:10.3390/nu13093159)
Supplement: Supplementary file 1 [file nutrients-13-03159-s001.zip › supplementary/Supplementary Figure S3. Animal protein associated taxa and ethnicity in the A) Metacardis and B ) HELIUS cohort.pdf]

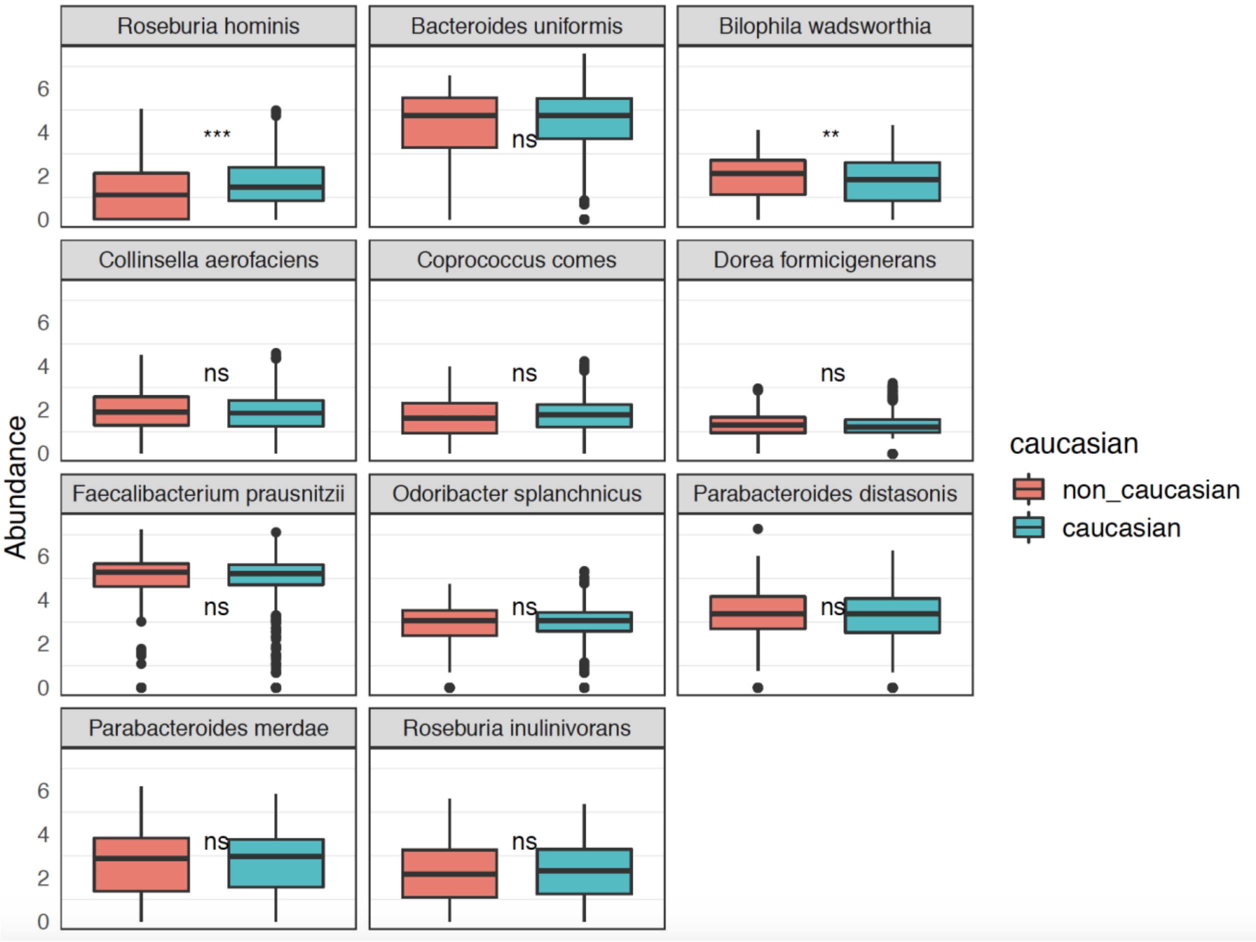

Supplementary Figure S3. Animal protein associated taxa and ethnicity in the A) MetaCardis and B) HELIUS cohort. *** p<0.001, ** p<0.01; T. test on log transformed abundance data
