# Supplementary material for: Protein Intake, Metabolic Status and the Gut Microbiota in Different Ethnicities: Results from Two Independent Cohorts
Source: Nutrients. 2021 Sep 10;13(9):3159. doi: 10.3390/nu13093159 (PMC8465773; doi:10.3390/nu13093159)
Supplement: Supplementary file 1 [file Supplementary Figure S4. Animal protein associated taxa and T2D in the A) MetaCardis and B)HELIUS.pdf › supplementary/Supplementary Figure S4. Animal protein associated taxa and T2D in the A) MetaCardis and B)HELIUS.pdf]

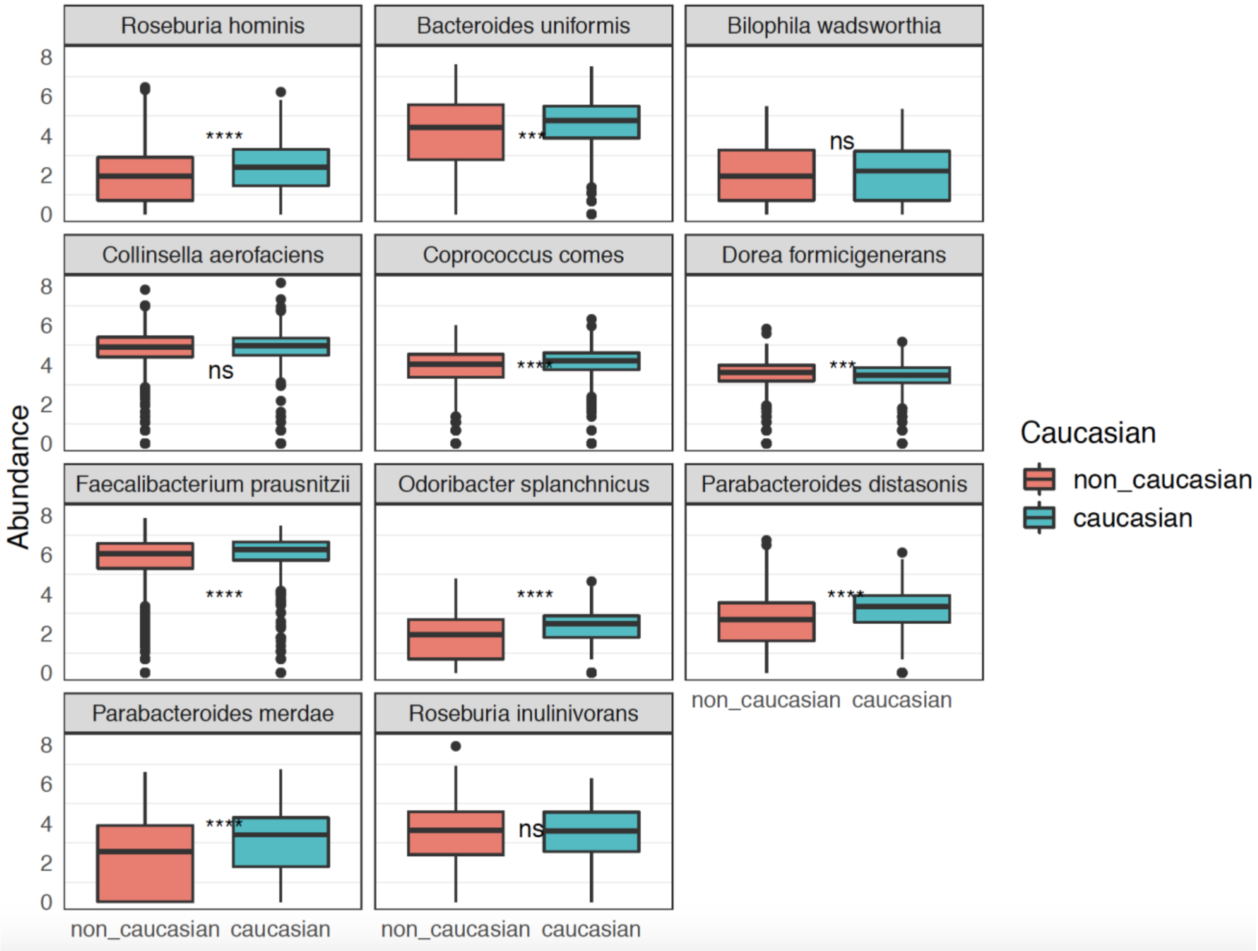


**Supplementary Figure S4.** Animal protein associated taxa and T2D in the A) MetaCardis and B ) HELIUS cohort .**** p<0.0001, *** p<0.001, ** p<0.01; T. test on log transformed abundance data
